# Exceptional therapeutic benefit of imatinib in cKIT‐mutated mucosal melanoma followed by a KIT mutation loss

**DOI:** 10.1111/ddg.15867

**Published:** 2025-10-25

**Authors:** Hannah Zillikens, Corinna Scheib, Vicky Peller, Christina Müller, Sebastian Bauer, Ina Pretzell, Klaus Griewank, Antje Sucker, Eva Hadaschik, Felicitas Guntrum, Sven Holger Baum, Alexander Roesch, Selma Ugurel, Dirk Schadendorf, Lisa Zimmer, Elisabeth Livingstone

**Affiliations:** ^1^ Klinik für Klinik für Dermatologie Venerologie und Allergologie Universitätsmedizin Essen; ^2^ Westdeutsches Tumorzentrum Universitätsklinikum Essen; ^3^ Westdeutsches Protonentherapiezentrum Universitätsklinikum Essen; ^4^ Mund‐ Kiefer‐und Gesichtschirurgie Helios St. Josefs Hospital Uerdingen

Dear Editors,

Mucosal melanoma accounts for approximately 1% of all melanomas and mainly affects the head and neck area (55%), with a focus on the nasal and paranasal sinuses.^1^, ^2^ Compared to cutaneous melanoma, it exhibits more aggressive growth behavior and a lower response to immune checkpoint inhibitors (ICI).^3^ *BRAF* mutations have been reported in 3% to 10% of cases, and NRAS mutations in 7% to 30%. In contrast, activating *cKIT* mutations occur 4% to 30% more frequently than in cutaneous melanomas (3%).^4^, ^5^

We report on a 64‐year‐old male patient with an R1‐resected mucosal melanoma of the left maxillary sinus, performed in December 2015, which was followed by proton radiation with 66 Gy. A year later, lymph node dissection was performed after detection of cervical lymph node metastasis on the left side (1 of 20 lymph nodes positive) (Figure 1). In January 2017, the patient presented for the first time with bipulmonary metastases at the Skin Tumor Center in Essen. Next‐generation sequencing (NGS) detected a *KIT* mutation in exon 11 (V559A) in the primary tissue and the lymph node metastasis. On the recommendation of the interdisciplinary skin tumor board, combined immune checkpoint inhibition with ipilimumab and nivolumab was initiated in February 2017, but had to be discontinued after a single dose due to side effects (grade 3 immune‐mediated colitis, grade 2 hepatitis, and grade 1 thyroiditis).

**FIGURE 1:**
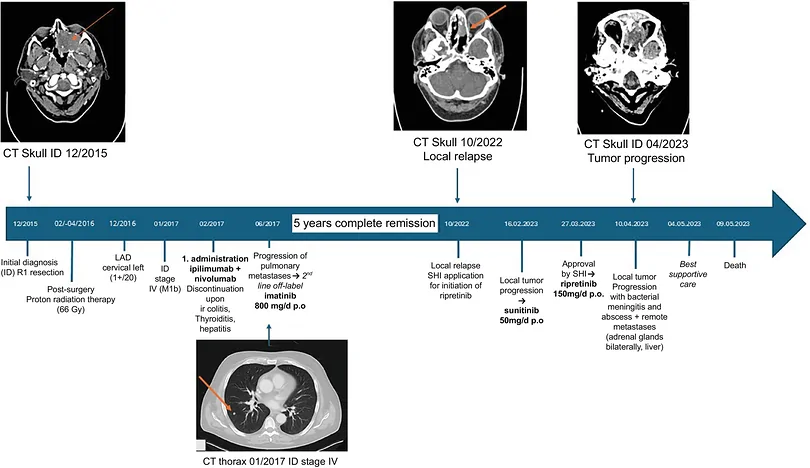
Timeline of the melanoma disease and treatment sequence.

In June 2017, treatment with the tyrosine kinase inhibitor imatinib at a dose of 800 mg/day was initiated for progressive lung metastases. After 6 weeks, a partial response was observed, and after 5 months, complete remission of the lung metastases was achieved, which persisted for 5 years.

A CT scan of the paranasal sinuses in October 2022, performed due to increasing headaches and a feeling of pressure behind the left eye, strengthened the suspicion of a local relapse, which was confirmed histologically. Given the inoperability and lack of radiation reserves, the molecular tumor board recommended an individual treatment attempt with the pan‐KIT inhibitor ripretinib following KIT resistance testing. Surprisingly, NGS analysis (GIST‐specific 31‐gene panel) of the local relapse no longer showed a *KIT* mutation. Due to the low tumor cell content of 15%, a sampling error was initially suspected and a new biopsy was performed. As symptoms increased, a bridging therapy with the multikinase inhibitor sunitinib at a dosage of 50 mg/day was started in February 2023, even before the new mutation analysis was available, as re‐exposure to anti‐PD‐1 inhibitors was not an option due to the lack of response in 2017. Because of the high treatment urgency, the health insurer ultimately approved the use of ripretinib despite the pending molecular pathology findings. Treatment with 150 mg/day was started in April 2023. However, the tumor continued to show rapid progression with infiltration of the frontobase and distant metastasis. The patient died one month later. Again, no *KIT* mutation was detected in the second biopsy.

Post‐mortem mutation analysis using a 611 gene pan‐cancer panel for the primary tumor, the cervical lymph node metastasis, and the local relapse confirmed the presence of the *KIT* mutation in the primary tumor and in the lymph node metastasis. In contrast, no *KIT* mutation was found in the local relapse tissue, although all other previously known mutations were confirmed (Table 1). A sample mix‐up, sequencing artifact and insufficient tumor cell content (80%) could thus be excluded.

**TABLE 1:** Gene mutations detected by NGS (611‑pan‑cancer gene panel) in tumor tissue of the primary tumor from December 2015, the lymph node metastasis from December 2016, and the local relapse from October 2022. Listed according to descending allele frequency of the gene mutations of the primary tumor.

Gene	Mutation	Primary tumor 12/2015 (Tumor cell content n.a.) Allele frequency	Lymph node metastasis 12/2016 (Tumor cell content 85 %) Allele frequency	Local relapse 11/2022 (Tumor cell content 80 %) Allele frequency
*KIT*	V559A	90 %	87 %	–
*ASXL2*	L278fs	80 %	72 %	58 %
*NOTCH2*	I1245T	62 %	52 %	47 %
*FANCC*	V60I	54 %	45 %	42 %
*MGA*	K542N	46 %	36 %	64 %
*CD274*	H233L	40 %	36 %	40 %
*TP63*	M510L	38 %	47 %	47 %
*DDR2*	V682I	30 %	33 %	44 %
*TSC2*	R1159Q	26 %	32 %	67 %
*BCLAF1*	T405R	26 %	30 %	8 %

*Abbr*.: n.a., not available

In mucosal melanomas, *cKIT* mutations are mostly localized in exon 11 (L576P) and 13 (K642E); exons 9, 17 and 18 are less frequently affected.^4^, ^5^ *cKIT* is a protooncogene that is partly responsible for the regulation of cell growth, cell differentiation and migration. KIT inhibitors are not approved for melanoma in Germany, but are used off‐label based on the results of phase II studies with objective response rates of 16% to 30%.^6^ A good response was seen above all for *KIT mutations* of exons 11 (L576P) and 13 (K642E).^5^, ^7^ Long‐term therapy usually leads to the development of resistance, which is often caused by *de novo* mutations in exon 17. A reduced response to available KIT inhibitors is known for these mutations, whereas a good response is often observed with ripretinib.^5^, ^6^, ^8^

Our recommendation of bridging treatment with the multikinase inhibitor sunitinib after the failure of imatinib was based on the combined inhibition of cKIT and VEGFR by sunitinib and the resulting increased inhibition of cell proliferation, metastasis, and neoangiogenesis. However, in this case the anticipated broader spectrum of efficacy did not materialize.

Ripretinib has the broadest spectrum of therapeutic activity to date due to its inhibition of KIT and platelet‐derived growth factor receptor A (PDGFRA), and it has been approved for oral tumor therapy in gastrointestinal stromal tumors (GIST) since May 2020.^9^ A recent phase I study by Janku et al. demonstrated a good response in patients with metastatic melanoma taking ripretinib 150 mg once daily and *cKIT* mutations in exon 11 and 17.^3^ Of the 26 patients involved, one achieved complete remission and five others achieved partial remission. The median progression‐free survival (mPFS) was 7.3 months, with exon 11 mutations at 10 months and exon 17 mutations at 13 months. Patients with prior KIT inhibitor therapy showed a response in 11% of cases, albeit significantly shorter (mPFS: 2.9 months) than KIT inhibitor‐naive patients (10.2 months). Ripretinib thus represents a therapeutic alternative in KIT inhibitor therapy failure and as second‐line therapy after immunotherapy, particularly for exon 17 mutations. Complete loss of the *KIT* mutation, as observed in the present case, has not been described for melanoma to date. Previous studies could only demonstrate reduced *KIT* expression in metastatic melanoma.^10^ The loss of *KIT* explains the lack of response to either sunitinib or ripretinib.

## CONFLICT OF INTEREST STATEMENT

All other authors declare no conflict of interest.

E.L. has served as a consultant and/or has received honoraria from Bristol Myers Squibb, Merck Sharp & Dohme, Novartis, Pierre Fabre, Sanofi, Sun Pharma, and Takeda and has received travel support from Bristol Myers Squibb, Pierre Fabre, Sun Pharma, and Novartis, outside the submitted work.

I.P. has served on the advisory board of BeiGene.

L.Z. has served as a consultant and has received honoraria from Bristol Myers Squibb, Merck Sharp & Dohme, Novartis, Pierre Fabre, Sanofi, and Sun Pharma and travel support from Merck Sharp & Dohme, Bristol Myers Squibb, Pierre Fabre, Sanofi, Sun Pharma, and Novartis, outside the submitted work.

S.U. declares research support from Bristol Myers Squibb and Merck Serono; speakers’ and advisory board honoraria from Bristol Myers Squibb, Merck Sharp & Dohme, Merck Serono, and Novartis; and meeting and travel support from Almirall, Bristol Myers Squibb, IGEA Clinical Biophysics, Merck Sharp & Dohme, Novartis, Pierre Fabre, and Sun Pharma, outside the submitted work.
